# Quantitative Measurement of Organic Acids in Tissues from Gastric Cancer Patients Indicates Increased Glucose Metabolism in Gastric Cancer

**DOI:** 10.1371/journal.pone.0098581

**Published:** 2014-06-09

**Authors:** Hoon Hur, Man Jeong Paik, Yi Xuan, Duc-Toan Nguyen, In-Hye Ham, Jisoo Yun, Yong Kwan Cho, Gwang Lee, Sang-Uk Han

**Affiliations:** 1 Department of Surgery, Ajou University School of Medicine, Suwon, Republic of Korea; 2 Institute for Gastric Cancer Mechanism, Ajou University School of Medicine, Suwon, Republic of Korea; 3 College of Pharmacy, Suncheon National University, Suncheon, Republic of Korea; 4 Department of Gastric Cancer and Soft Tissue Sarcoma, Fudan University Shanghai Cancer Center, Shanghai, People’s Republic of China; 5 National Institute of Drug Quality Control, Hoan Kiem, Ha Noi, Vietnam; 6 Department of Physiology, Ajou University School of Medicine, Suwon, Republic of Korea; University of Navarra, Spain

## Abstract

The levels of organic acids representing metabolic pathway end products are important indicators of physiological status, and may be associated with metabolic changes in cancer. The aim of this study is to investigate the levels of organic acids in cancerous and normal tissues from gastric cancer patients and to confirm the role of metabolic alterations in gastric carcinogenesis. Organic acids in normal and cancerous tissues from forty-five patients with gastric adenocarcinoma were investigated by gas chromatography-mass spectrometry in selected ion monitoring mode as methoxime/*tert*-butyldimethylsilyl derivatives. We analysed the significant differences in the levels of organic acids in normal and cancer tissues and investigated the correlation of these levels in cancer tissues with clinicopathological features. The levels of Krebs cycle components, including *α*-ketoglutaric acid, succinic acid, fumaric acid, malic acid and oxaloacetic acid, were significantly increased in cancer tissues compared to normal tissues. In addition, the levels of glycolytic products, including pyruvic acid and lactic acid, as well as the levels of ketone bodies, including 3-hydroxybutyric acid, were also significantly increased in cancer tissues compared to normal tissues. The levels of ketone bodies in cancer tissues with differentiated histology and in intestinal-type cancer tissues were significantly increased. The organic acid profiling analysis described here may be a generally useful clinical tool for understanding the complexity of metabolic events in gastric adenocarcinoma, and organic acids may have potential as metabolic markers for the future discovery of diagnostic and therapeutic modalities.

## Introduction

Although gastric cancer-related mortality has decreased, it is still the second most frequent cause of cancer-related death [1]. Many patients with gastric cancer are diagnosed at an advanced stage, and they have a high rate of recurrence after curative resection and a poor response to treatment [2], [3]. To improve the survival rate of gastric cancer, efforts have focused on the identification of patients with poor prognosis and new therapeutic modalities based on molecular mechanisms [4]. To date, genomic, epigenetic and proteomic studies have been used to elucidate molecular mechanism of gastric cancer, and to identify biomarkers associated with poor prognosis and poor response to treatment [4], [5]. These biomarkers could become target for treatment of patients with advanced gastric cancer [6]. However, the results of treatment for them are still unsatisfactory. It may be one of the reasons that the carcinogenic process of gastric cancers is complicated by the existence of multiple genetic variations and various external factors, such as *Helicobacter pylori* infection and salt ingestion [7]. Thus, the products of distinct metabolic pathways in malignant tumours that respond to complex genetic and environmental changes may be crucial biomarkers to predict the prognosis and to suggest therapeutic target in gastric cancer.

The important role of glucose metabolism in cancer cells is well established, and cancer cells exhibit increased glycolysis even under non-hypoxic conditions compared to normal cells [8]. Based on this property of cancer cells, 2-fluoro-2-deoxy-D-glucose positron emission tomography (FDG-PET) can be used to diagnose malignant tumours and predict the chemotherapeutic response [9], [10]. However, the mechanisms of aberrant glucose metabolism during carcinogenesis are not fully understood, complicating the use of members of this pathway as diagnostic tools and therapeutic targets. The quantitative measurement of organic acids (OAs), which are the end products of metabolic processes and may reflect cancer phenotypes, in tumour and non-tumour tissues of cancer patients may improve our understanding of the metabolic changes that occur in cancer. Organic acids may also be of use as novel biomarkers to predict disease progression, the response to treatment and prognosis. However, only a few reports on metabolic profiling of gastric cancer tissue have been published, and these reports have involved few patients [11], [12]. Although several methods, such as nuclear magnetic resonance (NMR) spectroscopy and mass spectrometry (MS), for the quantitative measurement of metabolites have been developed, gas chromatography (GC) coupled with mass spectrometry (MS) has become the gold standard for the analysis of small molecular weight metabolites due to its high sensitivity and reproducibility [13].

Thus, we hypothesised that metabolic profiling analysis using GC-MS for tumour and non-tumour gastric tissues may be beneficial for evaluating metabolic changes in gastric cancer. The differences in the levels of the metabolites between healthy and cancer tissue indicate the role that these pathways play in gastric carcinogenesis. In addition, for patients with cancers of various degrees of advancement and histological features, we attempted to classify the metabolic features according to the clinicopathological features of the gastric cancer.

## Materials and Methods

### Patients and Tissue Specimens

The study protocol was approved by the Institutional Review Board of Ajou University Hospital (Suwon, South Korea; AJIRB-MED-KSP-11-212), and written informed consent was obtained from all participating patients. From April to June 2010, 45 patients who were diagnosed with gastric adenocarcinoma by gastroscopic biopsy were enrolled. Computed tomography images of the abdomen and pelvis in addition to chest radiography and tumour markers were evaluated for clinical staging before the operation. Most of the patients had undergone gastric cancer surgery with curative intent, but six patients received palliative resection for bleeding and obstruction. Total or subtotal gastrectomy with proper lymph node dissection was performed, followed by reconstruction according to the treatment guidelines of the Japanese Gastric Cancer Association [14]. Immediately after surgical resection, tumour tissue and adjacent normal tissue were obtained from the 45 stomach cancer patients. The obtained tissues were immediately frozen in liquid nitrogen and stored at −80°C until use.

### Chemicals and Reagents

The following OA standards used in this study were purchased from Sigma-Aldrich (St. Louis, MO, USA): 3,4-dimethoxybenzoic acid as internal standard (IS), 3-hydroxybutyric acid, pyruvic acid, lactic acid, succinic acid, fumaric acid, oxaloacetic acid, *α*-ketoglutaric acid, malic acid, *cis*-aconitic acid, citric acid and isocitric acid. Acetoacetic acid was purchased from Tokyo Chemical Industry (Tokyo, Japan). Methoxyamine hydrochloride was also obtained from Sigma-Aldrich. N-methyl-N-(*tert*-butyldimethylsilyl) trifluoroacetamide (MTBSTFA)+1% *tert*-butyldimethylchlorosilane was obtained from Thermo Scientific (Bellefonte, PA, USA). Toluene, diethyl ether, ethyl acetate and dichloromethane (pesticide grade) were purchased from Kanto Chemical (Chuo-ku, Tokyo, Japan). Sodium hydroxide was supplied by Duksan (Ansan, South Korea), and sulphuric acid was purchased from Samchun Pure Chemical (Pyeongtaek, South Korea). All other chemicals were of analytical reagent grade.

### Quantitative Measurement of Metabolites Using the GC-MS Method

Derivatised samples were analysed in both scan and selected ion monitoring (SIM) mode with a 6890N gas chromatograph (Agilent Technologies, Santa Clara, CA, USA) interfaced to a 5975B mass-selective detector (70 eV, electron ionisation source; Agilent Technologies) as previously reported [15]. Briefly, the mass spectra were scanned in a range of 50–650 u at a rate of 0.99 scans/s. The temperatures of the injector, interface and ion source were 260, 300 and 230°C, respectively. An HP Ultra-2 (Agilent Technologies, Santa Clara, CA, USA) cross-linked capillary column coated with a 5% phenyl/95% methylpolysiloxane bonded phase (25 m×0.20 mm I.D.; 0.11 µm film thickness) was used for all analyses. Helium was used as the carrier gas at a flow rate of 0.5 mL/min in constant flow mode. Samples (1 µL) were introduced in split-injection mode (10∶1). The oven temperature was initially set at 100°C (2 min), increased to 250°C at a rate of 5°C/min and finally programmed to 300°C at a rate of 20°C/min (5 min). In SIM mode, three characteristic ions for each compound were used for peak confirmation, and one target ion was selected for quantification.

### Sample Preparation for Profiling Analysis of OA in Gastric Tissues

Distilled water was added to the gastric tissues, and the tissues were finely homogenised in an ice water bath with a T10 basic Ultra-Turrax® disperser (IKA-Werke GmbH & Co.KG, Staufen, Germany). Distilled water (500 µL), acetonitrile (500 µL) and IS (0.2 µg) were added to the aliquot of homogenate (equivalent to 10 mg of stomach tissue), and the mixture was vortexed (2 min) and centrifuged (14,000 rpm for 10 min) to precipitate proteins. The supernatant layer was adjusted to pH>12 with 5.0 M NaOH. The carbonyl groups were converted into methoxime (MO) derivatives by reaction with methoxyamine hydrochloride (1.0 mg) at 60°C for 30 min. The reaction mixture was then acidified to pH 1–2 with a 10% sulphuric acid solution saturated with sodium chloride and extracted with diethyl ether (3 mL) followed by ethyl acetate (2 mL). After addition of triethylamine (5 µL), the combined extracts were evaporated to dryness under a gentle stream of nitrogen (40°C). Toluene (20 µL) as the solvent and MTBSTFA (20 µL) as the silylation reagent were added to the residues, followed by heating at 60°C for 30 min to form methoxime/*tert*-butyldimethylsilyl derivatives for direct GC-SIM-MS analysis.

### Star Symbol Plotting

The concentrations of 12 OAs found in gastric cancer tissues were normalised to the corresponding means in the normal group, and each normalised value was plotted as a line radiating from a common central point. The far ends of lines were joined together to produce dodecagonal star patterns with Microsoft Excel as described elsewhere [16], [17].

### Statistical Analysis

All statistical analyses were performed with SPSS version 13.0 for Windows (IBM, Chicago, IL, USA). The levels of metabolites were compared between cancer tissues and normal tissues by the Wilcoxon matched pairs test. New variables, such as total glycolytic products, total Krebs cycle products and total ketone bodies, were created from the sum of the metabolites, which are the intermediates or end products in each pathway, and the differences in these variables in normal and cancer tissues were also evaluated by the Wilcoxon matched pairs test. The differences in the levels of the new variables as a function of the clinicopathological characteristics were analysed by the Mann-Whitney test. p<0.05 was considered statistically significant.

## Results

### Clinicopathological Characteristics and Measurement of OA Levels

**Figure 1:**
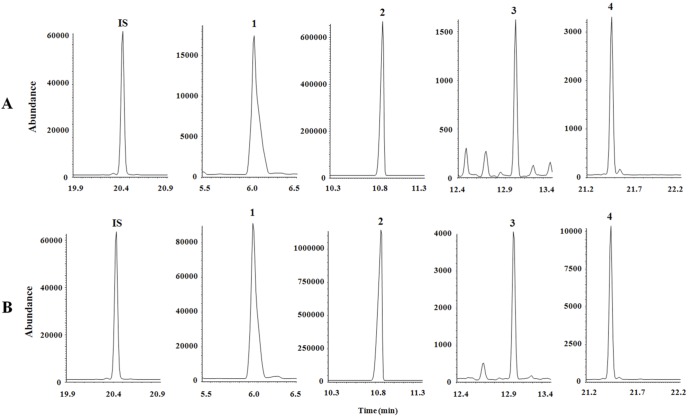
SIM chromatograms of organic acids in (A) normal and (B) cancer tissues. The following peaks are shown: IS, 3,4-dimethoxybenzoic acid; 1, pyruvic acid; 2, lactic acid; 3, 3-hydroxybutyric acid; and 4, *α*-ketoglutaric acid.

The mean age of the 45 enrolled patients was 61.8 years, and 71.1% of the patients were male. The proportion of patients with advanced gastric cancer was higher than that of patients with early gastric cancer (55.6%). Other clinicopathological factors are listed in Table 1. Representative SIM chromatograms of pyruvic, lactic, 3-hydroxybutyric and *α*-ketoglutaric acids in normal and cancer tissues are shown in Fig. 1.

**Table 1:** Clinicopathologic characteristics of enrolled patients (*n* = 45).

Variables	Number	Percentage (%)
Age (years)		
<65	25	55.6
≥65	20	44.4
Gender		
Male	32	71.1
Female	13	28.9
Diabetics		
Yes	9	20.0
No	36	80.0
Gastrectomy		
Total gastrectomy	7	15.6
Partial gastrectomy	38	84.4
Location		
Upper	6	13.3
Middle	15	33.3
Lower	24	53.3
T stage		
T1	20	44.4
T2	6	13.3
T3	9	20.0
T4	10	22.2
N stage		
N0	25	55.6
N1	4	8.9
N2	6	13.3
N3	10	22.2
M stage		
M0	39	86.7
M1	6	13.3
Differentiation		
Differentiated	24	53.3
Undifferentiated	21	46.7
Size		
<5 cm	24	53.3
≥5 cm	21	46.7
Lauren classification		
Intestinal	27	60.0
Mixed	8	17.8
Diffuse	10	22.2
*Helicobacter Pylori*		
Infection	13	28.9
No infection	32	71.1

### Comparison of OA Levels in Normal and Cancer Tissues

**Figure 2:**
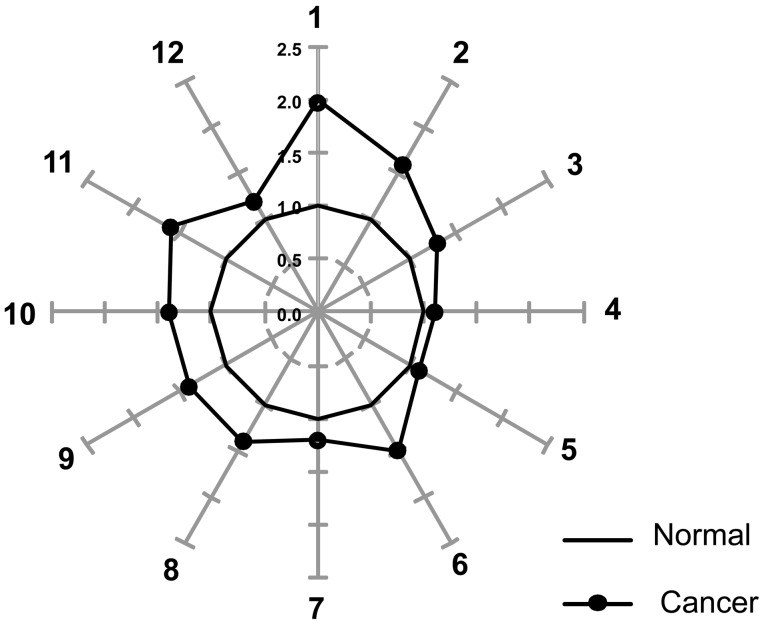
Star symbol plots of the mean data from cancer and normal tissues were constructed based on the levels of organic acids after normalisation to the corresponding normal mean values. The following rays are shown: 1, pyruvic acid; 2, lactic acid; 3, citric acid; 4, isocitric acid; 5, cis-aconitic acid; 6, *α*-ketoglutaric acid; 7, succinic acid; 8, fumaric acid; 9, malic acid; 10, oxaloacetic acid; 11, 3-hydroxybutyric acid; and 12, acetoacetic acid.

The mean values of the 12 OAs in the normal and cancer tissues are shown in Table 2. In the normal and cancerous tissues, lactic acid was the most abundant, followed by malic and pyruvic acids. However, normalisation of the mean value of OAs in cancer tissue to that in normal tissue revealed that pyruvic acid was significantly increased by 2-fold in cancer tissue. In addition, lactic and malic acid also displayed approximately 60 and 40% increases, respectively, in cancer tissue compared to normal tissue. The levels of *α*-ketoglutaric, succinic, fumaric, oxaloacetic and 3–hydroxybutyric acids were also significantly increased in cancer tissues compared to normal tissues, while no differences were observed in the levels of citric, isocitric, *cis*-aconitic and acetoacetic acids in normal and cancer tissues. When the normalised levels were used to construct star graphs composed of 12 rays, the differences between the cancer and normal tissues were more clear (Fig. 2). The star pattern of the cancer tissue was distorted, allowing it to be readily distinguished from the dodecagonal shape of normal tissue.

**Table 2:** Levels of Metabolites Related to Glucose Metabolism in Normal and Gastric Cancer Tissues.

Compounds	Normal tissue(ng/mg tissue)	Cancer tissue(ng/mg tissue)	Normalisedcancer tissue level	p-value*
Pyruvic acid	81.7±35.6	148.5±56.3	2.0±0.9	<0.001
Lactic acid	1933.9±751.9	2924.4±1249.5	1.6±0.8	<0.001
Citric acid	48.2±57.9	43.0±58.7	1.3±1.2	0.527
Isocitric acid	14.7±4.0	15.2±4.9	1.1±0.4	0.299
cis-Aconitic acid	4.3±3.9	3.7±2.9	1.1±0.5	0.639
*α*-ketoglutaric acid	8.0±4.2	10.8±5.3	1.5±0.7	0.001
Succinic acid	16.4±4.1	18.6±6.8	1.2±0.5	0.012
Fumaric acid	17.0±6.2	21.9±8.4	1.4±0.5	<0.001
Malic acid	242.4±102.8	321.9±146.7	1.4±0.6	<0.001
Oxaloacetic acid	10.8±4.2	13.9±4.7	1.4±0.5	<0.001
3-Hydroxybutyric acid	25.2±29.9	34.0±32.0	1.6±0.9	<0.001
Acetoacetic acid	21.3±9.0	23.2±10.6	1.2±0.7	0.516

All values are presented as the mean ± standard deviation.

*p-values were calculated by the Wilcoxon matched pairs test.

### Analysis of Values for Glycolytic Products, TCA Intermediates and Ketone Bodies

The total levels of glycolytic products were calculated from the sum of the levels of pyruvic and lactic acids in each tissue (Table 3). In addition, the total levels of Krebs cycle products were calculated from the sum of the levels of elevated metabolites related to the Krebs cycle, and the total levels of ketone bodies were calculated from the sum of the levels of 3-hydroxybutyric and acetoacetic acids in each tissue. The mean levels of the three calculated variables were significantly higher in cancer tissues than in normal tissues (p<0.001 for total glycolytic products; p<0.001 for total Krebs products; and p = 0.001 for total ketone bodies).

**Table 3:** Variables Calculated from the Measured Levels of Metabolites Related to Glycolytic Products, TCA Intermediates and Ketone Bodies.

Compounds	Normal tissue(ng/mg tissue)	Cancer tissue(ng/mg tissue)	Normalisedcancer tissue level	p-value**
Total glycolytic products*	2015.7±764.6	3072.9±1272.8	1.6±0.8	<0.001
Pyruvic acid				
Lactic acid				
Total TCA intermediates^1^	294.7±113.5	387.1±162.5	1.4±0.6	<0.001
* α*-ketoglutaric acid				
Succinic acid				
Fumaric acid				
Malic acid				
Oxaloacetic acid				
Total ketone bodies^1^	46.5±32.2	57.1±34.3	1.4±0.5	0.001
** 3**–Hydroxybutyric acid				
Acetoacetic acid				

*The sum of each metabolite is categorised according to the pathway for glucose metabolism.

**p-values were calculated by the Wilcoxon matched pairs test.

All values are presented as the mean ± standard deviation.

In addition, we analysed the levels of each variable in cancer tissues according to the clinicopathological factors of the participants, including age, gender, invasion depth, lymph node metastasis, size, Lauren classification and differentiation (Table 4). The levels of the three variables were relatively higher in cancer tissues with differentiated tumours than in those with undifferentiated tumours. However, only the difference in ketone bodies was significantly different (p = 0.009). In addition, the difference in the levels of ketone bodies among the three types of Lauren classification was also significant (p = 0.017).

**Table 4:** Comparisons of the Levels of Calculated Values According to Clinicopathological Features.

		Glycolytic products		TCA products		Ketone bodies	
	n	Mean ± S.D.(ng/mg tissue)	*P*-value*	Mean ± S.D.(ng/mg tissue)	*P*-value	Mean ± S.D.(ng/mg tissue)	*P*-value
Age (years)							
<65	25	3024.6±1398.3	0.664	387.2±169.4	0.802	52.6±30.5	0.568
≥65	20	3133.4±1129.2		387.1±158.0		62.8±38.7	
Gender							
Male	32	3124.2±1430.9	0.822	383.3±166.8	0.783	57.2±34.8	0.764
Female	13	2946.9±791.9		396.5±157.6		57.0±34.5	
Depth							
EGCa	20	3171.3±1432.1	0.493	403.7±175.4	0.337	50.3±28.1	0.385
AGCa	25	2994.2±1154.2		373.9±153.8		62.6±38.3	
Node							
No metastasis	25	3062.0±1329.0	0.767	411.6±171.1	0.315	56.0±31.1	0.891
Metastasis	20	3086.7±1245.3		356.5±149.8		58.5±38.8	
Size							
<5 cm	24	3256.3±1459.1	0.295	412.1±169.0	0.172	54.6±31.4	0.964
≥5 cm	21	2863.4±1014.7		358.6±153.9		60.0±38.0	
Lauren							
Intestinal	27	3360.6±1276.5	0.281	425.9±158.8	0.136	65.3±34.0	0.017
Mixed	8	2789.7±1257.3		358.5±169.2		30.7±12.6	
Diffuse	10	2522.9±1152.5		307.6±148.1		56.2±38.5	
Differentiation							
Differentiated	24	3429.1±1322.0	0.088	428.0±166.0	0.097	68.5±34.7	0.009
Undifferentiated	21	2665.9±1108.3		340.5±148.8		44.2±29.6	

EGCa = early gastric cancer. AGCa = advanced gastric cancer.

*p-values were calculated by the Mann-Whitney test.

All values are presented as the mean ± standard deviation.

## Discussion

To the best of our knowledge, this is the first demonstration of altered OA levels in paired cancer and normal tissue samples obtained from 45 patients with gastric adenocarcinoma. Although the generated data are complex, the differences in OA levels between normal and cancer tissues correlated with the levels of metabolites, including glycolytic products. The increased level of ketone bodies in cancer tissues was significantly related to the histological features of gastric cancer.

Aerobic glycolysis in malignant tumours was well-described more than 60 years ago by Warburg and is known as “the Warburg effect” [8]. The altered glycolytic pathway in malignant tumours is activated by the upregulation of several enzymes, such as glucose tranporter-1 (GLUT-1) and hexokinase-2. In gastric cancer, positive immunohistochemical staining for GLUT-1 has been related to tumour invasion and lymph node metastasis [18], [19]. However, detection of the expression of metabolic pathway-related molecules has not led to the development of novel diagnostic or therapeutic tools. The quantitative measurement of metabolic products from the glycolytic pathway may yield more sensitive markers than the expression of enzymes in gastric cancer. Previous reports have demonstrated that the measurement of metabolites may be a possible means of evaluating the metabolic switch, such as aerobic glycolysis to non-mitochondrial oxidative phosphorylation, in a malignant tumour [13], [20]. In the present study, the levels of pyruvic and lactic acids, which are the metabolites related to the glycolytic pathway, were significantly elevated in cancer tissues compared to normal tissues. In addition, the star symbol plots, which were based on the levels of 12 OAs after normalisation to the corresponding normal tissue samples, were found to be effective for the visual identification of cancer tissue samples due to their distorted dodecagonal patterns. Although normal tissue samples were found to be adequate as a control pattern for cancer tissue samples, there is an urgent need for large-scale studies of OAs to clarify the significance of the changes in OA levels in cancer tissues from patients with gastric adenocarcinoma. The elevation of many OAs, which may result from a cascade of aerobic glycolysis, indicates an altered metabolism in gastric cancer tissues.

The concentrations of metabolites, which are small molecules present in human tissues or fluids, can be measured to evaluate biological abnormalities in cancer tissue. Analysis of malignant tumours compared with normal tissues has become a sensitive tool for cancer research due to the development of metabolomic technology, which enables the quantification and identification of metabolomes [13]. Several reports have shown that products of metabolic pathways, such as phosphocholine and glycerophosphocholine, are elevated in breast cancer tissues compared to benign or normal tissues [21]–[23]. Other studies on prostate and brain cancers have also reported increased glycolytic products in tumour tissues, but all other studies have applied NMR techniques to measure the levels of metabolites in human tissues. Here, we used a more sensitive and selective method, GC-MS, to measure the levels of metabolites in paired normal and cancerous gastric tissues. GC-MS can identify more than 100 compounds from a small amount of human tissue, and Chan et al. previously reported the use of GC-MS to measure metabolic products in biopsied colorectal cancer and normal tissues [24]. In the present study, we performed OA profiling analysis with approximately 10 mg of tissue. Although we measured OA levels using tissues obtained during surgical resection, preoperative measurement may be clinically more meaningful to determine the treatment modality. Because tissues weighing more than 5 mg can be obtained by gastroscopic biopsy, it is possible to apply our technique on biopsied specimens. Moreover, we kept the interval time between resection and freezing in the operation room as short as possible to reduce the bias from metabolic distortion following tissue ischemia during surgical resection. Therefore, GC-MS-based profiling of metabolites related to the metabolism of glucose in resected or biopsied tissues may represent a sensitive technique to monitor the changes in glucose metabolism in cancer tissue.

To maintain homeostasis in normal cells, intermediate metabolites, such as citric acid, oxaloacetic acid and *α*-ketoglutaric acid, which are involved in the Krebs cycle, are used for the synthesis of fatty acids, nucleic acids and amino acids. Meanwhile, metabolic changes in cancer cells reduce the production of acetyl CoA from pyruvate, the end product of glycolysis, due to the dysfunction of pyruvate dehydrogenase, which may lead to insufficient acetyl CoA as the precursor for the Krebs cycle [25]. To supply anabolic precursors for tumour growth, concomitant mechanisms, such as glutaminolysis, are likely activated, resulting in changes in the Krebs cycle [26]. Thus, it was anticipated that the levels of intermediate metabolites from the Krebs cycle would be different in cancer tissues and normal tissues (Table 2). Among the intermediates of the glucose metabolism pathway, the levels of lactic acid, which is the final end product of glycolysis, were highest in both cancer tissues and normal tissues. In the first three steps of the Krebs cycle, citric, *cis*-aconitic acid and isocitric acid are generated from acetyl CoA, which is a source of oxidative phosphorylation in mitochondria. The levels of these metabolites did not differ appreciably between normal and cancer tissues. The Krebs cycle can operate using another source of input, such as glutamine, and *α*-ketoglutaric acid is the first product of glutaminolysis. The levels of the products that are generated after *α*-ketoglutaric acid, including succinic, fumaric, malic and oxaloacetic acids, were significantly higher in cancer tissue than in normal tissue.

**Figure 3:**
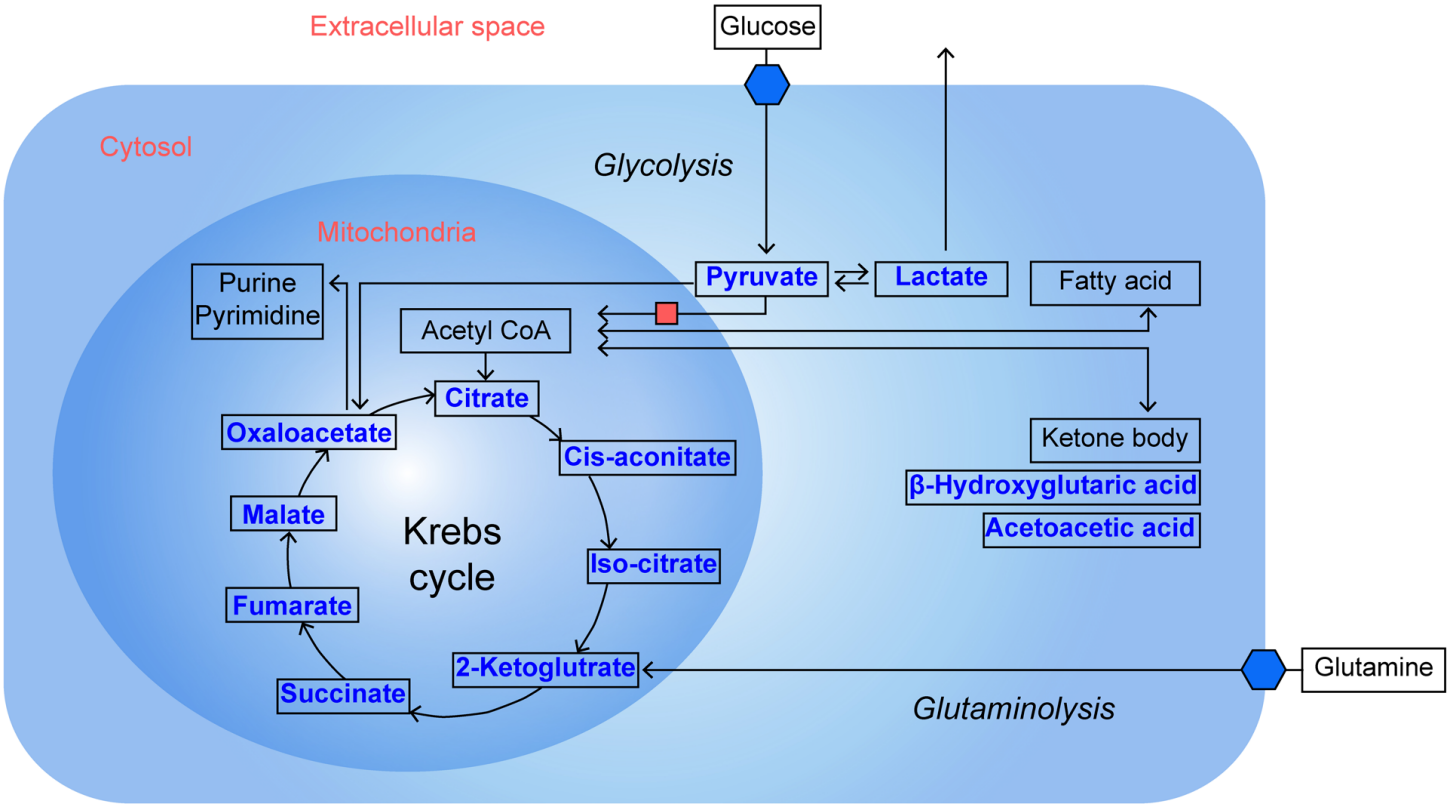
Schematic presentation of organic acids that are increased in gastric cancer tissues compared to normal gastric tissue. This well-known pathway describes the inhibition of the conversion of pyruvate to acetyl CoA, alteration of the TCA cycle, increased glutaminolysis and ketone body production. Organic acids within the box were measured for both cancer and normal tissues in this study. *represents significantly increased levels of OAs in gastric cancer tissues.

The uptake of ketone bodies in tumour cells has been observed in response to hypoxic conditions in head and neck cancer [27]. Increasing utilisation of ketone bodies can contribute to energy production in a malignant tumour, although this likely represents a small fraction of energy production compared to the uptake of glucose. An *in vivo* investigation has shown that changes in the 3-hydroxybutylic acid/acetoacetate ratio may be a sensitive marker of tumour progression [28]. Even increased ketone in a tumour could enhance several genes that were related to the prognosis of patients with breast cancers. [29] These previous results suggested the possibility of ketone measurement as a biomarker to predict the survival of the patients with malignant tumours. In present study, as several intermediates of glucose metabolism were increased in cancer tissue, 3-hydroxybutyric acid, a kind of ketone body, was also significantly increased in tumour tissues compared to normal gastric tissues. Schematic presentations of OA levels including glycolytic intermittents and ketone bodies according to metabolic pathway are shown in Fig. 3.

In present study, the level of total ketone bodies in tumour tissues was significantly increased in gastric cancers of differentiated histology and intestinal type. The carcinogenesis of gastric cancer differs according to histological type. Intestinal gastric cancer is caused by infection with *Helicobacter pylori*, subsequent gastritis and tissue regeneration [30]. Although the phenotypes of metabolic change depending on histological type have not been completely characterised, the accuracy of FDG-PET based on the abnormality of glucose metabolism depends on differentiation in gastric cancer [31]. Thus, diagnostic and therapeutic techniques based on metabolite measurements may be applicable to specific histological types of gastric cancer. Prior to clinical application, additional studies are needed on the correlation between metabolite levels and clinical outcomes, such as the survival rate. However, recent studies have been hampered by several limitations, including low patient numbers and the duration of follow-up. Further clinical studies should be undertaken to confirm the role of OA profiling as a diagnostic modality or the use of metabolic biomarkers to predict disease prognosis.

In conclusion, we demonstrated that OA levels in paired cancer and normal tissue samples obtained from patients with gastric adenocarcinoma exhibit significant metabolic differences. These results may be important to understanding how OA changes are related to glucose metabolism. The OA profiling analysis in the present study may be a generally useful clinical tool for understanding the complexity of metabolic events in gastric adenocarcinoma. Moreover, this method may be a useful technique for the future discovery of gastric cancer-specific biomarkers for diagnostic and therapeutic strategies.
